# Healthcare Resource Utilization Associated with Leukopenia and Neutropenia in Kidney Transplant Recipients Receiving Valganciclovir in the United States

**DOI:** 10.36469/001c.125097

**Published:** 2025-01-29

**Authors:** Qinghua Li, Vladimir Turzhitsky, Pamela Moise, Harry Jin, Kaylen Brzozowski, Irina Kolobova

**Affiliations:** 1 Merck & Co., Inc., Rahway, New Jersey, USA; 2 TriNetX, Cambridge, Massachusetts, USA; 3 TriNetX, Cambridge, Massachusetts, USA

**Keywords:** kidney transplantation, neutropenia, leukopenia, valganciclovir, myelosuppression, healthcare utilization

## Abstract

**Background:** Cytomegalovirus prophylaxis in kidney transplant recipients (KTRs) is limited by post-transplant neutropenia and leukopenia (PTN/PTL). Despite its clinical significance, the healthcare resource utilization (HCRU) related to PTN/PTL remains poorly characterized. **Objective:** To evaluate HCRU among KTRs taking valganciclovir during their first year post-transplant. **Methods:** Using TriNetX Dataworks-USA, a federated, de-identified electronic medical record database, we identified adult KTRs who underwent their first kidney transplant from January 2012 to September 2020. All eligible patients were followed for 1 year. PTN/PTL was defined as absolute neutrophil count less than 1000/μL or white blood cell count less than 3500/μL. Multivariable logistic/Poisson regression models were used to assess the association between PTN/PTL and various HCRU types. **Results:** A total of 8791 KTRs were identified, of whom 6219 (70.7%) developed PTN/PTL at a mean of 5.7 months post-transplantation. Hospitalizations, rehospitalizations, emergency room visits, outpatient appointments, packed red blood cell transfusions, and granulocyte-colony stimulating factor administration were more prevalent among KTRs with PTN/PTL (61.1% vs 49.5%, 24.5% vs 14.1%, 35.2% vs 28.9%, 30.4 vs 26.2 visits, 22.3% vs 17.6%, 23.4% vs 2.2%, respectively; P < .001). Adjusted analyses confirmed that PTN/PTL correlated with increased HCRU across all categories. **Conclusions:** KTRs who developed PTN/PTL had significantly higher HCRU. Further studies are needed to evaluate strategies addressing PTN/PTL for KTRs.

## INTRODUCTION

Approximately 37 million Americans have kidney disease, the tenth leading cause of death in the United States.^1,2^ Kidney transplantation is considered the optimal treatment for patients with end-stage renal disease (ESRD).^3^ As of April 2023, over 87 000 adult patients were waitlisted for a kidney transplant.^4^ The number of kidney transplantations in the United States has significantly increased since 2015, exceeding 25 000 kidney transplants in 2022.^3,4^ As of late 2018, there were 229 887 kidney transplant recipients (KTRs) with a functioning kidney allograft.^3^

KTRs are at high risk for the development of opportunistic infections due to the use of induction and maintenance immunosuppression, and these infections pose a significant cause of morbidity and mortality, particularly during the first year post-transplant. One common complication in kidney transplantation is cytomegalovirus (CMV) infection, which frequently complicates the course after kidney transplantation and can have some very serious consequences. Prior studies reported that among KTRs, the incidence of CMV infection or disease varies from less than 10% to over 50%.^5–13^ To prevent progression to CMV disease in KTRs, most transplantation centers administer universal prophylaxis with valganciclovir/ganciclovir.^14,15^ Current guidelines recommend 6 months of post-transplant prophylaxis for CMV seronegative KTRs who receive an organ from a CMV seropositive donor (D+/R-), and 3 months when the KTR has a preexisting CMV-specific immunity (ie, for CMV R+ KTRs). While oral valganciclovir is the current standard of care for prophylaxis of CMV disease in D+/R- and R+ KTRs, myelosuppression, specifically post-transplant neutropenia (PTN) and post-transplant leukopenia (PTL),^16,17^ limits its use and may lead to worse clinical outcomes. Other drugs, such as mycophenolate, antithymocyte globulin (ATG), tacrolimus, and cotrimoxazole, have also been found to result in myelotoxicity. Such complications further add significant clinical burdens, including escalating the complexity of medical decision-making by providers, and increasing healthcare resource utilization (HCRU) in KTRs.^18–20^

A recent systematic review reported PTN/PTL to be common and associated with acute graft rejection, immunosuppressant dose modifications, and opportunistic infections.^19^ However, data on the HCRU associated with PTN/PTL among KTRs are limited. This study aimed to evaluate HCRU among KTRs taking valganciclovir who developed PTN/PTL during their first-year post-transplant.

## METHODS

### Study Design and Data Source

This was a retrospective observational cohort study using de-identified data from TriNetX Dataworks-USA, a federated network with real-time updates of de-identified electronic medical records of approximately 86 million patients from 54 healthcare organizations across the United States. This study used only de-identified patient records and was exempt from institutional review board approval.

**Figure 1.**
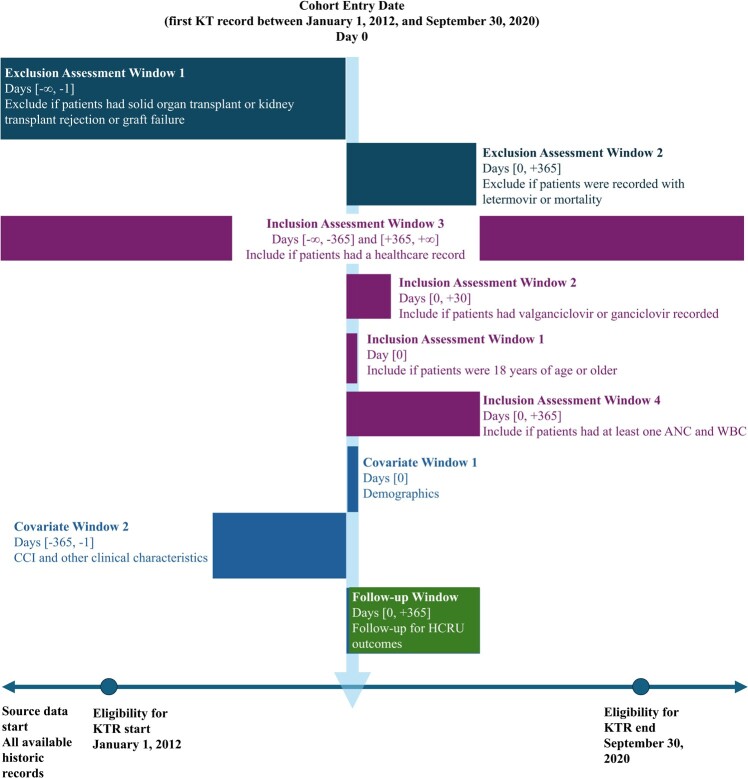
Study Design Abbreviations: ANC, absolute neutrophil count; CCI, Charlson comorbidity index; HCRU, healthcare resource utilization; KT, kidney transplant; KTR, kidney transplant recipient; WBC, white blood cell count. Note: Exclusion, inclusion, covariate, and follow-up windows are described.

Adult patients who had their first kidney transplant between January 1, 2012, and September 30, 2020, were included in the study. The date of their first kidney transplantation was the index date of the analysis. The baseline period was defined as from 1 year before the index date to the day before the index date. Patients were followed for up to 1 year post-index. The study design is represented in **Figure 1**.

### Study Population

This study included adult KTRs who met the following criteria:

1. Received their first kidney transplantation between January 1, 2012, and September 30, 2020
2. Were 18 years or older at the time of their kidney transplant
3. Had at least 1 record of receiving valganciclovir or ganciclovir within 30 days post-transplant
4. Had healthcare records at least 1 year before the index date and at least 1 year following the index date
5. Had at least 1 laboratory test result for absolute neutrophil count (ANC) and white blood cell count (WBC) during the follow-up period

Patients with evidence of solid-organ transplant at any time before their index kidney transplantation, a history of kidney transplant rejection or graft failure before their index kidney transplantation, or any record of letermovir prescription or mortality during the follow-up period were excluded.

Kidney transplantation was identified by a CPT or ICD-10-PCS procedure record of kidney transplant. Laboratory records of ANC and WBC were identified by LOINC laboratory records. Solid organ transplantation prior to index was identified by a CPT, ICD-10-PCS, ICD-10-CM, or ICD-9-CM record of any solid organ (lung, liver, heart, stomach, intestine, pancreas) transplantation. The list of medical codes used to identify patients in this study and the following variables can be found in **Supplementary Table S1**.

### Variables

**Definitions of post-transplant neutropenia and leukopenia**: PTN/PTL was defined as the development of neutropenia and/or leukopenia from the time of kidney transplantation until the end of the 1-year follow-up period. PTN was defined as having at least 1 ANC <1000 cells/μL. PTL was defined as having at least 1 laboratory result for a WBC <3500 cells/μL. These thresholds are commonly used in studies of PTN/PTL.^19^

**Baseline characteristics**: All baseline characteristics measured were identified within 1 year before the index kidney transplantation. Sociodemographic data included age at the time of transplant, sex, and race. The Quan adaptation of the Charlson Comorbidity Index (CCI) was calculated based on diagnoses recorded during the baseline.^21–23^ Renal disease was excluded from this study’s calculation of the CCI due to the eligibility criteria requiring all patients to have received a kidney transplant. Other clinical characteristics included myocardial infarction, peripheral vascular disease, cerebrovascular disease, peptic ulcer disease, and dialysis received during the baseline.

**Immunosuppression and other drugs**: Induction immunosuppressive agents included antithymocyte globulin (ATG), alemtuzumab, and basiliximab.^24^ Maintenance immunosuppressive agents assessed included calcineurin inhibitors (tacrolimus or cyclosporine), corticosteroids, mycophenolate mofetil, azathioprine, and mTOR inhibitors (sirolimus or everolimus).^25^ Trimethoprim-sulfamethoxazole, which is used for prophylaxis of *Pneumocystis jirovecii* and well known for causing neutropenia/leukopenia, was also evaluated.^26^

**Healthcare resource utilization outcomes**: HCRU outcomes included the proportion of patients utilizing a resource and the total number of hospital admissions, emergency room (ER) visits, outpatient visits, granulocyte-colony stimulating factor (G-CSF) use, and post-transplant packed red blood cell (PRBC) transfusions. All HCRU outcomes were captured from the index kidney transplant date to the end of the follow-up period.

**Hospital admissions and emergency room visits**: Inpatient admissions and ER visits were defined by their respective procedure or administrative records for inpatient hospital care, or emergency department care, respectively, on unique dates excluding the index hospitalization period. ER visits excluded any emergency department visit resulting in an inpatient admission. Rehospitalization was defined as a second inpatient admission within 30 days of discharge from any unique hospitalization.

**Outpatient visits**: Outpatient visits were defined by procedure or administrative records for care in an outpatient or ambulatory setting. An outpatient visit was identified by querying records within the follow-up period for an outpatient encounter on unique dates of service.

**G-CSF administration and packed red blood cell transfusions**: G-CSF was evaluated during the follow-up period and included the number of G-CSF prescriptions and time to the first instance of G-CSF administration. PRBC transfusions were identified through procedural records.

### Statistical Analysis

**Descriptive analysis**: Baseline characteristics and HCRU outcomes were compared between patients with PTN/PTL and those without. Continuous measures were reported as mean and SD, or median and interquartile range (IQR). Categorical measures were presented with frequency (count) and percentage. Comparisons between those with and without PTN/PTL were conducted using *t*-tests for continuous variables and c^2^ tests for categorical variables. A *P* value of less than .05 was considered statistically significant for all univariate tests.

**Adjusted analyses**: We estimated multivariable regression models to examine the association between PTN/PTL and HCRU. The logistic regression model was fit for outcomes to define if a patient utilized a specific type of healthcare, and Poisson model for number of utilizations. We ran univariate analyses between each HCRU outcome and candidate covariates; candidate covariates were selected based on clinical considerations of factors medically important to HCRU outcomes as well as factors that were significantly associated with PTN/PTL based on descriptive analyses. Factors significantly associated with each outcome at the *P* < .2 level were included in the final multivariable regression for that outcome. Covariates in the adjusted regressions included age group (18-24, 25-34, 35-44, 45-64, ≥65 years); race (White, Black or African American, Other, and Unknown); sex (male/female), Quan adaptation of the CCI (continuous–increments of 1); diabetes during the baseline (yes/no); number of dialysis sessions during the baseline (continuous–increments of 1); induction antithymocyte globulin use (yes/no); induction basiliximab use (yes/no); initial maintenance cyclosporin use (yes/no); initial maintenance tacrolimus use (yes/no); initial maintenance mycophenolate mofetil use (yes/no), and trimethoprim-sulfamethoxazole use (yes/no).

All data management and analysis were performed with SAS version 9.4 (SAS).

## RESULTS

During the study period, 8791 KTRs were identified by attrition criteria included in **Table 1**. Baseline demographics and characteristics of the cohort with and without PTN/PTL are shown in **Table 2**. The mean (SD) age of all KTRs was 52.8 (13.3) years; 41.6% were white, 32.6% were black, and 59.3% were male. Among all KTRs, 2149 (24.4%) had PTN, 6127 (69.7%) had PTL, and 6219 (70.7%) had either PTN or PTL. Kidney transplant recipients with PTN/PTL were more likely to be aged 45 to 64 years (*P*=.002), female (*P* < .001), and have a higher BMI (*P* < .001) than patients without PTN/PTL.

**Table 1.** Attrition Table

**Inclusion/Exclusion Criteria**	**N**	**% from Previous Row**
KT between Jan. 1, 2013, and Sept. 30, 2020	25 900	–
Aged ≥18 years at index	25 220	97.4
Healthcare record >365 days prior to index	19 903	78.9
Valganciclovir or ganciclovir recorded during 1 month on or after index	15 677	78.8
ANC and WBC tests during 365 days after index	12 678	80.9
No evidence of solid organ transplant prior to index	11 731	92.5
No history of KT prior to index	11 000	93.8
No history of KT rejection or graft failure	9407	85.5
No letermovir record on or during 365 days after index	9368	99.6
Healthcare record >365 days following index	8791	93.8

Abbreviations: ANC, absolute neutrophil count; KT, kidney transplant; WBC, white blood cells.

**Table 2.** Demographic and Baseline Clinical Characteristics

**Baseline Characteristics^a^**	**All KTR**	**KTR With PTN/PTL^b^**	**KTR Without PTN/PTL^b^**	***P* Value**
Total, n (%)	8791	6219 (70.7)	2572 (29.3)	
Age, years				
Mean (SD)	52.8 (13.3)	52.9 (13.2)	52.6 (13.4)	.45
Age group, n (%)				.002
18-24	192 (2.2)	150 (2.4)	42 (1.6)	
25-34	794 (9.0)	537 (8.6)	257 (10.0)	
35-44	1346 (15.3)	914 (14.7)	432 (16.8)	
45-64	4606 (52.4)	3312 (53.3)	1294 (50.3)	
65+	1853 (21.1)	1306 (21.0)	547 (21.3)	
Race, n (%)				<.001
White	3660 (41.6)	2619 (42.1)	1041 (40.5)	
Black or African American	2864 (32.6)	2120 (34.1)	744 (28.9)	
Other (American Indian/Alaska Native, Asian, Native Hawaiian or other Pacific Islander)	523 (5.9)	367 (5.9)	156 (6.1)	
Unknown	1744 (19.8)	1113 (17.9)	631 (24.5)	
Sex				.021
Male	5211 (59.3)	3638 (58.5)	1573 (61.2)	
Female	3580 (40.7)	2581 (41.5)	999 (38.8)	
BMI at index				
Mean (SD)	30.6 (13.7)	31.57 (15.0)	27.44 (7.5)	<.001
Median, IQR (25th-75th)	28.6 (23.8-⁠33.9)	28.75 (23.8-⁠34.4)	28.1 (23.7-⁠32.6)	
BMI group at (closest to) index, n (%)				
Patients with BMI information	4937 (56.2)	3712 (59.7)	1225 (47.6)	
<18.5	457 (5.2)	348 (5.6)	109 (4.2)	.009
18.5-24.9	912 (10.4)	681 (11.0)	231 (9.0)	.006
25-29.9	1618 (18.4)	1165 (18.7)	453 (17.6)	.22
≥30	1950 (22.2)	1518 (24.4)	432 (16.8)	<.001
Calculated BMI	4321 (49.2)	3304 (53.1)	1017 (39.5)	<.001
BMI from diagnosis codes	616 (7.0)	408 (6.6)	208 (8.1)	
BMI not available	3854 (43.8)	2507 (40.3)	1347 (52.4)	
Quan adaptation of Charlson Comorbidity Index scorec				
Mean, SD	1.2 (1.5)	1.2 (1.5)	1.2 (1.5)	.95
Median, IQR (25th-75th)	1 (0-2)	1 (0-2)	1 (0-2)	
Quan CCI comorbidities, n (%)^c,d^				
Congestive heart failure	1031 (11.7)	731 (11.8)	300 (11.7)	.90
Chronic pulmonary disease	623 (7.1)	450 (7.2)	173 (6.7)	.40
Mild liver disease	1782 (20.3)	1236 (19.9)	546 (21.2)	.15
Diabetes with chronic complications	2205 (25.1)	1573 (25.3)	632 (24.6)	.48
Other comorbidities^d^				
Peripheral vascular disease	729 (8.3)	515 (8.3)	214 (8.3)	.95
Dialysis, n (%)	1492 (17.0)	1059 (17.0)	433 (16.8)	.83
Mean No. (SD) of dialysis sessions	16.1 (59.1)	17.8 (62.5)	12.1 (49.5)	.07
Induction immunosuppressants				
Antithymocyte globulin, n (%)	3825 (43.5)	2813 (45.2)	1012 (39.3)	<.001
Mean time to initiation, days (SD)	0.6 (1.4)	0.5 (1.4)	0.6 (1.5)	.29
Basiliximab, n (%)	1388 (15.8)	797 (12.8)	591 (23.0)	<.001
Mean time to initiation, days (SD)	0.6 (1.6)	0.6 (1.6)	0.6 (1.5)	.82
Alemtuzumab, n (%)	558 (6.3)	364 (5.9)	194 (7.5)	.003
Mean time to initiation, days (SD)	0.0 (0.5)	0.0 (0.5)	0.0 (0.5)	.98
Maintenance immunosuppressants				
Calcineurin inhibitors (cyclosporine/tacrolimus), n (%)	8648 (98.4)	6111 (98.3)	2537 (98.6)	.21
Mean time to initiation, days (SD)	1.1 (1.6)	1.1 (1.7)	1.1 (1.5)	.18
Tacrolimus, n (%)	8628 (98.1)	6100 (98.1)	2528 (98.3)	.53
Mean time to initiation, days (SD)	1.1 (1.6)	1.1 (1.7)	1.1 (1.5)	.13
Corticosteroid therapy, n (%)	8766 (99.7)	6202 (99.7)	2564 (99.7)	.76
Mean time to initiation, days (SD)	0.2 (1.3)	0.2 (1.3)	0.1 (1.3)	.06
Mycophenolate mofetil, n (%)	6845 (77.9)	4967 (79.9)	1878 (73.0)	<.001
Mean time to initiation, days (SD)	0.4 (1.2)	0.4 (1.3)	0.4 (1.1)	.46
Trimethoprim-sulfamethoxazole, n (%)	7848 (89.3)	5573 (89.6)	2275 (88.5)	.11
Mean time to initiation, days (SD)	1.3 (1.8)	1.3 (1.8)	1.4 (1.7)	.14

Abbreviations: IQR, interquartile range; KTR, kidney transplant recipient; PTN/PTL, post-transplant neutropenia and leukopenia.

^a^ Baseline characteristics recorded during the 365 days prior to index transplantation.

^b^ Neutropenia defined as absolute neutrophil count <1000/μL, and <500 ANC/μL during the 365-day follow-up period (includes index date).

^c^ Charlson Comorbidity Index calculation followed the Quan adaptation and included the following variables: congestive heart failure, dementia, chronic pulmonary disease, rheumatologic disease, mild liver disease, diabetes with chronic complications, hemiplegia or paraplegia, any malignancy (including leukemia and lymphoma, moderate or severe liver disease, metastatic solid tumor, and HIV/AIDS. Renal disease was excluded from the calculation due to eligibility criteria of patients having kidney transplants.

^d^ Comorbidities and induction immunosuppressive medications are presented if they had ≥5% prevalence among the total cohort. Those with >5% prevalence include dementia, rheumatologic disease, hemiplegia/paraplegia, any malignancy, moderate/severe liver disease, metastatic solid tumor, AIDS/HIV, myocardial infarction, cerebrovascular disease, peptic ulcer disease, cyclosporine, azathioprine, sirolimus, and everolimus.

The most frequently utilized agents for immunosuppressive therapy included ATG, corticosteroids, calcineurin inhibitors, and mycophenolate mofetil, with prevalence rates ranging from 43.5% to 99.7%. Additionally, trimethoprim-sulfamethoxazole use was prevalent in 89.3% of KTRs. The utilization of ATG and mycophenolate mofetil was found to be significantly higher in KTRs with PTN/PTL compared with those without PTN/PTL (*P* < .001).

The descriptive analysis of the HCRU outcomes is shown in **Table 3**. Mean (SD) and median (IQR) onset of PTN/PTL were 5.7 (3.6) and 5.2 (3.1-8.6) months after kidney transplantation, respectively. Kidney transplant recipients with PTN/PTL were more likely to have at least one inpatient admission in the 1-year follow-up than those without (61.1% vs 49.5%, *P* < .001), and they tended to have more inpatient admissions (mean, 3.7 vs 2.6, *P* < .001). Similarly, a higher proportion of KTRs with PTN/PTL had a rehospitalization as compared with those without PTN/PTL (24.5% vs 14.1%, *P* < .001), and the average number of rehospitalizations was larger for those with PTN/PTL (4.5 vs 3.2, *P* < .001). The prevalence of ER visits among KTR with PTN/PTL was greater than those without (35.2% vs 28.9%, *P* < .001). The majority (99.8%) of all KTRs had an outpatient visit during the follow-up period, and the mean number of outpatient visits were greater among those with PTN/PTL than those without (30.4 vs 26.2, *P* < .001).

**Table 3.** Healthcare-Related Utilization During Follow-up

**HCRU ^a^**	**All KTR**	**KTR With PTN/PTL^b^**	**KTR Without PTN/PTL^b^**	***P* Value**
Total, n (%)	8791 (100)	6219 (70.7)	2572 (29.3)	
Months of follow-up, mean (SD)	47.3 (24.8)	47.4 (24.6)	47.2 (25.5)	.81
Months to neutropenia or leukopenia, mean (SD)			5.7 (3.6)	–
Months to neutropenia or leukopenia, median (IQR)			5.2 (3.1-8.6)	–
Inpatient admissions (excluding IP stays on index date)				
Any, n (%)	5070 (57.7)	3797 (61.1)	1273 (49.5)	<.001
No. of events, mean (SD)	3.4 (5.6)	3.7 (6.1	2.6 (3.5)	<.001
No. of events, median (IQR)	2.0 (1-3)	2.0 (1-3)	2.0 (1-3)	
Inpatient rehospitalization within 30 days ^c^				
Any, n (%)	1887 (21.5)	1524 (24.5)	363 (14.1)	<.001
No. of events, mean (SD)	4.2 (8.0)	4.5 (8.5)	3.2 (5.1)	<.001
Emergency room visits				
Any, n (%)	2931 (33.3)	2187 (35.2)	744 (28.9)	<.001
No. of events, mean (SD)	1.9 (1.6)	1.9 (1.5)	1.9 (1.7)	.78
Outpatient visits				
Any, n (%)	8773 (99.8)	6212 (99.9)	2561 (99.6)	.003
No. of events, mean (SD)	34.6 (29.3)	35.6 (3.4)	32.1 (26.2)	<.001
Packed red blood cell transfusions, n (%)	1838 (20.9)	1386 (22.3)	452 (17.6)	<.001
Packed red blood cell transfusions, mean (SD)	1.7 (1.7)	1.8 (1.9)	1.5 (1.3)	<.001
Packed red blood cell transfusions, median (IQR)	1.0 (1-2)	1.0 (1-2)	1.0 (1-1)	
Granulocyte colony-stimulating factor, n (%)	1511 (17.2)	1454 (23.4)	57 (2.2)	<.001
No. of records, mean (SD)	8.5 (18.3)	8.7 (18.6)	3.6 (4.6)	.04
Time to first record, mean (SD)	117.0 (72.3)	116.9 (72.2)	121.3 (73.7)	.65

Abbreviations: IQR, interquartile range; KTR, kidney transplant recipient; PTN/PTL, post-transplant neutropenia and leukopenia.

^a^ HCRU assessed during the 365 days after index transplantation.

^b^ Neutropenia defined as ANC <1000/μL, and <500 ANC/μL during the 365 days follow-up period (includes index date).

^c^ Rehospitalization includes rehospitalization within 30 days of any prior hospitalization.

PRBC transfusions were more prevalent among KTRs with PTN/PTL than those without (22.3% vs 17.6%, *P* < .001). G-CSF was recorded in 23.4% of patients with PTN/PTL, compared with 2.2% without (*P* < .001). Moreover, those who developed PTN/PTL had a higher number of G-CSF records, with 8.7 prescriptions on average compared with 3.6 (*P*=.038). Mean (SD) time to first record of G-CSF was 116.9 (72.2) days and 121.3 (73.7) days among those with and without PTN/PTL, respectively. Among those with PTN/PTL who used G-CSF after transplant, median (IQR) time to PTN/PTL was 4.9 (3.2-7.6) months.

**Figure 2.**
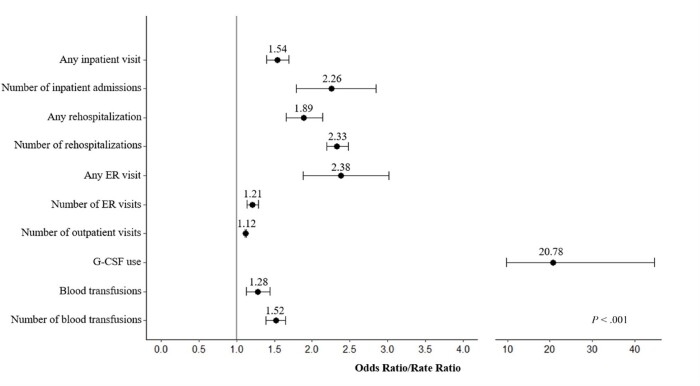
Association Between Post-transplant Neutropenia and Leukopenia and HCRU in Year 1 Post-Kidney Transplantation Abbreviations: ER, emergency room; G-CSF, granulocyte-colony stimulating factor; HCRU, healthcare resource utilization; OR, odds ratio; PTN/PTL, post-transplant neutropenia and leukopenia; RR, rate ratio. Note: Ratios and 95% confidence intervals for PTN/PTL are presented from outcomes in multivariate analysis in **Tables S2- S4**. Rate ratios are plotted for outcomes that begin with “Number” (Poisson regression) and odds ratios are plotted for other remaining outcomes (logistic regression). All ratios have significance at *P* < .001. Rehospitalization includes rehospitalization within 30 days of any prior hospitalization. Blood transfusions were packed red blood cell transfusions.

**Supplementary Tables S2, S3, and S4** present the multivariate regression results, displaying selected covariates for each model alongside corresponding regression outcomes. After controlling for demographic and clinical characteristics, PTN/PTL was found consistently associated with all types of HCRU, also illustrated in **Figure 2**. Kidney transplant recipients with PTN/PTL had 1.5 (95% CI: 1.4-1.7) times the odds of any hospitalization versus those without, and the rate of hospitalizations was 2.3 (95% CI: 1.8-2.9) times greater among those with PTN/PTL vs those without. Similarly, significant associations with PTN/PTL status were observed in overall rehospitalizations (OR = 1.9; CI: 1.7-2.1) and the number of rehospitalizations (RR =2 .3; 95% CI: 2.2-2.5). PTN/PTL also demonstrated associations with ER visits, with an OR of 2.4 (95% CI: 1.9-3.0) for any ER visit and an RR of 1.2 (95% CI: 1.1-2.3) for the number of ER visits. PTN/PTL was associated with the number of outpatient visits, with an RR of 1.1 (95% CI: 1.1-1.1). Significant associations were found between PTN/PTL and G-CSF administration, with an OR of 20.8 (95% CI: 9.7-44.5). Similarly, overall PTN/PTL was associated with an increased risk of PRBC transfusions, with an OR of 1.3 (95% CI: 1.1-1.4), as was the number of PTN/PTL-related PRBC transfusions, with an RR of 1.5 (95% CI: 1.4-1.7).

## DISCUSSION

This retrospective observational cohort study of 8791 KTRs receiving valganciclovir shows that PTN/PTL is associated with significantly higher HCRU in the first year post-kidney transplantation. Kidney transplant recipients with PTN/PTL had significantly more hospitalizations, ER visits, and outpatient visits compared with those without PTN/PTL. Those with PTN/PTL were also more likely to receive at least 1 PRBC transfusion and had a greater number of PRBC transfusions than those without PTN/PTL, and the same pattern was observed for the utilization of G-CSF.

The prevalence of PTN/PTL identified in this study was similar or greater than estimates from previous studies,^27,28^ and this study is one of the few to assess multiple measures of HCRU among KTRs receiving valganciclovir for CMV prophylaxis. Research conducted using registry-linked Medicare data found that among patients with D+/R- serostatus, 61% experienced rehospitalization.^29^ Additionally, 51% of these patients had an ER visit, 99% had an outpatient visit, and 15% received G-CSF after kidney transplant; 85.8% of these patients received CMV prophylaxis with valganciclovir.^24^ Another single-institution study found that 22% of patients had a neutropenia- or leukopenia-related hospitalization, and 62% were treated with G-CSF.^27^

While common approaches to managing myelotoxicity may be stepwise, with the most common first step involving a reduction (or withdrawal of) mycophenolic acid derivatives, followed by withdrawal of trimethoprim/sulfamethoxazole, valganciclovir withdrawal constitutes a third step in the approach to manage myelotoxicity, leading to discontinuation in 10% to 20%.^30^ A recent study by Vincenti et al showed neutropenia led to higher rates of discontinuations of valganciclovir and mycophenolic acid.^31^ Furthermore, this study found neutropenia in high-risk KTRs was also associated with more rejection and higher HCRU, including hospitalizations and use of G-CSF. This multiprong approach is associated with increased HCRU due to additional monitoring, and the increased risk of infections and graft complications that occur with the reduction of valganciclovir and immunosuppressants.^28–30,32^ Although difficult to quantify, provider attempts to address the development of PTN/PTL adds considerable complexity to post-transplant management, opening up the opportunity for errors in medical decision-making and indirectly leading to allograft dysfunction, and other collateral iatrogenic complications. The considerable increase in HCRU among KTRs experiencing PTN/PTL compared with those not experiencing PTN/PTL in this study suggests that these complications should be minimized to the extent possible to prevent complications in post-transplant management. The real-world nature of this data highlights this need in clinical practice. Novel CMV prophylaxis agents should also be evaluated for their improved toxicity profiles and their ability to reduce HCRU.

This study adds new insight to understanding PTN/PTL among KTRs receiving valganciclovir for CMV prophylaxis and HCRU associated with PTN/PTL for KTRs. The underlying data set (TriNetX Dataworks-USA Network) is a large, de-identified EMR-based network of multiple healthcare organizations across the United States. The data set is geographically and generally demographically representative and is constituted by academic and community medical centers. Single-center studies and other studies of smaller scale are often limited by sample size, especially considering that kidney transplantation is generally an infrequent procedure among individual organizations. EMR data provided a great opportunity to address the objective of evaluating HCRU outcomes since it contains de-identified, patient-level encounter records including laboratory data.

### Limitations

This study had limitations. CMV-seropositivity represents a recognized risk factor for adverse outcomes, particularly evident in cases with a positive donor and negative recipient (D+/R-), which is associated with the highest risk. However, it is important to note that the present database lacks the capacity to capture such disparities in serological status, thus limiting the assessment of its impact on the findings. Although the ability to access laboratory results was a positive attribute to standardize study cohorts, the clinical relevance of laboratory thresholds differs between institutions and patients. Laboratory measures are also subject to provider decisions on the testing frequency and availability of laboratory results in the EMR, for example, when commercial laboratories are used to determine ANC. As with studies utilizing EMR data, this investigation was subject to inherent limitations, including data entry errors and missing data. While de-identified EMR data provided this study with the best level of detail and insight to address key aims, only care provided by the contributing healthcare organizations was captured in this study, which could lead to underestimation of HCRU, the incidence of PTN/PTL, and other characteristics/events evaluated in this study. This analysis followed an intention-to-treat approach where events occurring after index were not accounted for as potential confounders, and outcomes assessed were all-cause since they were not directly associated by principal record of the KT as the cause. Post-index events such as additional transplants may have influenced results, although unlikely in a differential manor between cohorts. All-cause HCRU represents the overall burden after KT, but KT- and PTN/PTL-related visits were not specifically discussed in this study; we assumed the vast majority of healthcare utilization was due to KT. Resource use for PTN/PTL is difficult to capture as visits related to PTN/PTL would likely be recorded as a primary visit for KT. KTRs must be followed closely, and this study assumes that any healthcare use after KT would involve some discussion and care related to the transplant. Lastly, the generalizability of this study should be contextualized to a US-based healthcare-seeking population presenting to typically large institutions; standalone clinics and smaller medical centers are not commonly captured in this data set and may involve separate HCRU implications.

## CONCLUSION

To our knowledge, this is the largest study to comprehensively assess HCRU among KTRs receiving valganciclovir for CMV prophylaxis and among those experiencing PTN/PTL. While PTN/PTL is manageable, patients and providers should be aware of the significant healthcare burden these complications introduce to KTRs receiving CMV prophylaxis in the first year post-transplant. Furthermore, increased hospitalizations, outpatient visits, and complexities in medical decision- making the development of PTN/PTL is a possible “Pandora’s box” of iatrogenic complications that should be minimized if possible.

### Disclosures

Q.L., V.T., P.M., and I.K. were employees of Merck Sharp & Dohme LLC, a subsidiary of Merck & Co., Inc., Rahway, New Jersey, USA. K.B. and H.J. were employees of TriNetX, LLC at the time of this study.

### Ethics Statement

Ethical approval and informed consent were waived due to the retrospective nature of the study.

## Supplementary Material

Table S1

Online Supplementary Material
