# Supplementary material for: Healthcare Resource Utilization Associated with Leukopenia and Neutropenia in Kidney Transplant Recipients Receiving Valganciclovir in the United States
Source: J Health Econ Outcomes Res. 2025 Jan 29;12(1):22–9. doi: 10.36469/001c.125097 (PMC11784900; doi:10.36469/001c.125097)
Supplement: Online Supplementary Material [file jheor_2025_12_1_125097_263882.pdf]

Journal of Health Economics and Outcomes Research

Online Supplementary Material

Healthcare Resource Utilization Associated with Leukopenia and Neutropenia in Kidney Transplant Recipients Receiving Valganciclovir in the United States. *JHEOR*. 2025;12(1):22-29. doi:10.36469/jheor.2025.125097

Table S1: Medical Codes Used to Define Kidney Transplant Cohort, Covariates, and HCRU

Table S2: Adjusted Rate of Inpatient HCRU During Follow-up

Table S3: Adjusted Rate of Outpatient, Emergency, and G-CSF HCRU During Follow-Up

Table S4: Adjusted Rate of Blood Transfusion HCRU During Follow-Up

This supplementary material has been provided by the authors to give readers additional information about their work.

This is an open-access article distributed under the terms of the Creative Commons Attribution 4.0 International License (CCBY-4.0). View this license's legal deed at http://creativecommons.org/licenses/by/4.0 and legal code at http://creativecommons.org/licenses/by/4.0/legalcode for more information.

Table S2. Adjusted Rate of Inpatient HCRU During Follow-up

	Inpatient[a]											
**Characteristic**[b]	**Any Inpatient Visit**			**No. of Inpatient Visits**			**Any Rehospitalization**			**No. of Rehospitalizations**		
	**Odds Ratio**	**95% CI**	***P* Value**	**Rate Ratio**	**95% CI**	***P* Value**	**Odds Ratio**	**95% CI**	***P* Value**	**Rate Ratio**	**95% CI**	***P* Value**
Neutropenia or leukopenia[c]	1.5	(1.4-1.7)	<.001	2.3	(1.8-2.9)	<.001	1.9	(1.7-2.1)	<.001	2.3	(2.2-2.5)	<.001
Age group[d]												
18-24 y	1.3	(0.9-1.7)	.13	1.3	(0.8-2.2)	.29	1.2	(0.9-1.8)	.23	0.9	(0.8-1.1)	.17
25-34 y	1.0	(0.8-1.1)	.61	0.8	(0.6-1.2)	.25	0.9	(0.7-1.1)	.16	1.1	(1.0-1.2)	.004
34-44 y	1.1	(0.9-1.2)	.33	0.9	(0.7-1.2)	.48	0.9	(0.8-1.1)	.27	1.0	(0.9-1.1)	.93
65+ y	1.1	(1.0-1.2)	.11	1.3	(1.0-1.6)	.03	1.2	(1.0-1.3)	.02	1.2	(1.1-1.2)	<.001
Race[d]												
Black or African American	1.2	(1.0-1.2)	.15	1.4	(1.2-1.8)	<.001	1.2	(1.0-1.3)	.008	1.0	(0.9-1.0)	.09
Other[e]	0.7	(0.6-0.9)	.002	1.8	(1.3-2.6)	<.001	0.7	(0.6-0.9)	.009	0.4	(0.4-0.5)	<.001
Unknown	0.5	(0.5-0.6)	<.001	2.0	(1.6-2.5)	<.001	0.5	(0.4-0.6)	<.001	0.4	(0.4-0.4)	<.001
Sex												
Female	1.1	(1.0-1.2)	.14	1.0	(0.8-1.2)	.81	1.1	(1.0-1.2)	.09	–	–	–
CCI	1.0	(1.0-1.0)	.41	1.1	(1.1-1.2)	<.001	–	–	–	0.9	(0.9-0.9)	<.001
Diabetes prior to transplant	1.1	(1.0-1.3)	.02	1.1	(0.9-1.3)	.46	1.1	(1.0-1.3)	.04	1.1	(1.0-1.1)	.009
Dialysis prior to transplant	–	–	–	1.0	(1.0-1.0)	.024	–	–	–	1.0	(1.0-1.0)	<.001
Induction immunosuppressants												
Antithymocyte globulin	–	–	–	–	–	–	1.0	(0.9-1.1)	.92	1.0	(1.0-1.1)	.09
Basiliximab	0.9	(0.8-1.1)	.23	–	–	–	–	–	–	–	–	–
Maintenance immunosuppressants												
Cyclosporin	–	–	–	1.3	(0.9-2.0)	.22	1.3	(0.9-1.7)	.13	1.5	(1.4-1.7)	<.001
Tacrolimus	–	–	–	1.0	(0.6-1.5)	.89				0.9	(0.8-0.9)	.001
Mycophenolate mofetil	1.3	(0.6-2.7)	.58	0.7	(0.1-5.0)	.72	1.4	(0.6-3.1)	.46	–	–	–
Trimethoprim-sulfamethoxazole	0.8	(0.4-1.7)	.50	1.7	(0.2-11.8)	.62	0.7	(0.3-1.7)	.48	–	–	–

Abbreviations: CI, confidence interval; CCI, Charlson Comorbidity Index; HCRU, healthcare resource utilization; PTN/PTL, post-transplant neutropenia and leukopenia.
[a] HCRU assessed during the 365 days after index transplant. Ratios were adjusted by characteristics and baseline variables with univariate association (P<.20) with HCRU outcomes, including categorical age, race, sex, PTN/PTL, continuous CCI, diabetes prior to kidney transplant, dialysis prior to kidney transplant, induction antithymocyte globulin and basiliximab, and maintenance cyclosporin, tacrolimus, mycophenolate mofetil, and trimethoprim-sulfamethoxazole.
[b] Variables without univariate association with a given HCRU outcome were not included in the logistic or Poisson regression and therefore are not presented with an adjusted result.
[c] Neutropenia defined as ANC <1000/µL, and <500 ANC/µL during the 365 days follow-up period (includes index date).
[d] Comparator groups across categories included age 45-64, white race, and male sex.
[e] "Other" race includes American Indian/Alaska Native, Asian, Native Hawaiian or other Pacific Islander.

Table S3. Adjusted Rate of Outpatient, Emergency, and G-CSF HCRU During Follow-Up

Characteristic[b]	Outpatient, ER, and G-CSF Use[a]											
	Any ER Visit			No. of ER Visits			No. of Outpatient Visits			G-CSF Use		
	Odds Ratio	95% CI	*P* Value	Rate Ratio	95% CI	*P* Value	Odds Ratio	95% CI	*P* Value	Rate Ratio	95% CI	*P* Value
Neutropenia or leukopenia[c]	2.4	(1.9-3.0)	<.001	1.2	(1.1-1.3)	<.001	1.1	(1.1-1.1)	<.001	20.8	(9.7-44.5)	<.001
Age group[d]												
18-24 y	1.4	(0.8-2.4)	.24	1.7	(1.4-1.9)	<.001	1.1	(1.1-1.1)	<.001	0.7	(0.3-1.5)	.36
25-34 y	0.8	(0.6-1.2)	.23	1.3	(1.2-1.5)	<.001	1.0	(0.9-1.0)	<.001	1.3	(0.8-2.2)	.24
34-44 y	0.9	(0.7-1.2)	.47	1.2	(1.1-1.3)	<.001	1.0	(1.0-1.0)	<.001	1.5	(1.0-2.1)	.06
65+ y	1.3	(1.0-1.6)	.02	1.1	(1.0-1.2)	.009	1.1	(1.1-1.1)	<.001	1.0	(0.6-1.4)	.78
Race[d]												
Black or African American	1.5	(1.2-1.8)	<.001	1.3	(1.2-1.4)	<.001	1.0	(1.0-1.1)	<.001	0.9	(0.6-1.2)	.33
Other[e]	1.9	(1.3-2.8)	<.001	0.8	(0.7-0.9)	<.001	1.0	(1.0-1.0)	.52	1.0	(0.5-1.8)	.95
Unknown	2.1	(1.7-2.6)	<.001	1.2	(1.1-1.3)	<.001	1.3	(1.3-1.3)	<.001	1.4	(1.0-2.1)	.07
Sex												
Female	1.0	(0.8-1.2)	.78	1.2	(1.1-1.3)	<.001	1.0	(1.0-1.0)	<.001	0.9	(0.7-1.3)	.64
CCI	1.1	(1.1-1.2)	<.001	1.1	(1.1-1.1)	<.001	1.0	(1.0-1.0)	<.001	1.1	(1.0-1.2)	.12
Diabetes prior to transplant	1.1	(0.9-1.3)	.45	1.3	(1.2-1.3)	<.001	1.1	(1.1-1.1)	<.001	1.8	(1.0-1.6)	.30
Dialysis prior to transplant	1.0	(1.0-1.0)	.01	1.0	(1.0-1.0)	<.001	1.0	(1.0-1.0)	<.001	1.0	(1.0-1.0)	.07
Induction immunosuppressants												
Antithymocyte globulin	–	–	–	0.9	(0.8-1.0)	.001	1.0	(1.0-1.0)	.004	–	–	–
Basiliximab	–	–	–	1.1	(1.1-1.3)	.003	1.1	(1.1-1.1)	<.001	–	–	–
Maintenance immunosuppressants												
Cyclosporin	–	–	–	1.1	(0.9-1.2)	.50	0.9	(0.9-0.9)	<.001	–	–	–
Tacrolimus	1.0	(0.6-1.5)	.87	0.9	(0.8-1.0)	.11	–	–	–	–	–	–
Mycophenolate mofetil	–	–	–	1.4	(1.0-2.1)	.09	1.1	(1.0-1.2)	.009	–	–	–
Trimethoprim-sulfamethoxazole	1.2	(1.0-1.5)	.09	0.7	(0.5-1.0)	.08	0.9	(0.8-0.9)	<.001	–	–	–

Abbreviations: CI, confidence interval; CCI, Charlson Comorbidity Index; HCRU, healthcare resource utilization; PTN/PTL, post-transplant neutropenia and leukopenia.
[a] HCRU assessed during the 365 days after index transplant. Ratios were adjusted by characteristics and baseline variables with univariate association (*P*<.20) with HCRU outcomes, including categorical age, race, sex, PTN/PTL, continuous CCI, diabetes prior to kidney transplant, dialysis prior to kidney transplant, induction antithymocyte globulin and basiliximab, and maintenance cyclosporin, tacrolimus, mycophenolate mofetil, and trimethoprim-sulfamethoxazole.
[b] Variables without univariate association with a given HCRU outcome were not included in the logistic or Poisson regression and therefore are not presented with an adjusted result.
[c] Neutropenia defined as ANC <1000/µL, and <500 ANC/µL during the 365 days follow-up period (includes index date).
[d] Comparator groups across categories included age 45-64, white race, and male sex.
[e] "Other" race includes American Indian/Alaska Native, Asian, Native Hawaiian or other Pacific Islander.

Table S4. Adjusted Rate of Blood Transfusion HCRU During Follow-Up

Characteristic[b]	Blood Transfusion[a]					
	Any Blood Transfusion			No. of Blood Transfusions		
	Odds Ratio	95% CI	*P* Value	Odds Ratio	95% CI	*P* Value
Neutropenia or leukopenia[c]	1.3	(1.1-1.4)	<.001	1.5	(1.4-1.7)	<.001
Age group[d]						
18-24 y	1.3	(0.9-1.9)	.13	1.2	(1.0-1.5)	.13
25-34 y						
34-44 y	0.8	(0.7-1.0)	.04	0.7	(0.6-0.8)	<.001
65+ y	1.6	(1.4-1.8)	<.001	1.4	(1.3-1.6)	<.001
Race[d]						
White	–	–	–	–	–	–
Black or African American	1.5	(1.3-1.7)	<.001	1.4	(1.3-1.5)	<.001
Other[e]	1.3	(1.1-1.7)	.01	1.1	(0.9-1.3)	.32
Unknown	1.0	(0.8-1.1)	.65	0.9	(0.8-1.0)	.07
Sex						
Female	1.5	(1.3-1.6)	<.001	1.4	(1.3-1.5)	<.001
CCI	1.0	(1.0-1.0)	.99	1.0	(1.0-1.0)	.99
Diabetes prior to transplant	1.1	(1.0-1.2)	.19	–	–	–
Dialysis prior to transplant	1.0	(1.0-1.0)	.58	1.0	(1.0-1.0)	<.001
Induction immunosuppressants						
Antithymocyte globulin	–	–	–	–	–	–
Basiliximab	1.0	(0.8-1.1)	.05	–	–	–
Maintenance immunosuppressants						
Cyclosporin	–	–	–	–	–	–
Tacrolimus	–	–	–	–	–	–
Mycophenolate mofetil	–	–	–	0.9	(0.8-1.0)	.10
Trimethoprim-sulfamethoxazole	1.4	(0.6-3.3)	.40	1.1	(0.6-2.0)	.71
Trimethoprim-sulfamethoxazole	0.6	(0.3-1.4)	.27	0.9	(0.5-1.6)	.65

Abbreviations: CI, confidence interval; CCI, Charlson Comorbidity Index; HCRU, healthcare resource utilization; PTN/PTL, post-transplant neutropenia and leukopenia.

[a] HCRU assessed during the 365 days after index transplant. Ratios were adjusted by characteristics and baseline variables with univariate association ($P < .20$). with HCRU outcomes, including categorical age, race, sex, PTN/PTL, continuous CCI, diabetes prior to kidney transplant, dialysis prior to kidney transplant, induction anti-thymocyte globulin and basiliximab, and maintenance cyclosporin, tacrolimus, mycophenolate mofetil, and trimethoprim-sulfamethoxazole

[b] Variables without univariate association with a given HCRU outcome were not included in the logistic or Poisson regression and therefore are not presented with an adjusted result.

[c] Neutropenia defined as ANC <1000/μL, and <500 ANC/μL during the 365 days follow-up period (includes index date).

[d] Comparator groups across categories included age 45-64, white race, and male sex.

[e] "Other" race includes American Indian/Alaska Native, Asian, Native Hawaiian or other Pacific Islander.
